# Determination of Postprandial Glycemic Responses by Continuous Glucose Monitoring in a Real-World Setting

**DOI:** 10.3390/nu11102305

**Published:** 2019-09-27

**Authors:** Martin Röhling, Tobias Martin, Meinolf Wonnemann, Martin Kragl, Horst Harald Klein, Lutz Heinemann, Stephan Martin, Kerstin Kempf

**Affiliations:** 1 West-German Centre of Diabetes and Health, Düsseldorf Catholic Hospital Group, Hohensandweg 37, 40591 Düsseldorf, Germany; Stephan.Martin@uni-duesseldorf.de (S.M.); kerstin.kempf@wdgz.de (K.K.); 2 Faculty of Medicine, Ruhr-University Bochum, 44801 Bochum, Germany; tobias.martin130297@gmail.com (T.M.); harald.klein@bergmannsheil.de (H.H.K.); 3 Bionorica SE, 92318 Neumarkt, Germany; meinolf.wonnemann@bionorica.de (M.W.); martin.kragl@bionorica.de (M.K.); 4 Science Consulting in Diabetes GmbH, 41462 Neuss, Germany; lutz.heinemann@profil.com; 5 Faculty of Medicine, Heinrich Heine University Düsseldorf, 40225 Düsseldorf, Germany

**Keywords:** postprandial glucose response, continuous glucose monitoring

## Abstract

Background: Self-monitoring of blood glucose using capillary glucose testing (C) has a number of shortcomings compared to continuous glucose monitoring (CGM). We aimed to compare these two methods and used blood glucose measurements in venous blood (IV) as a reference. Postprandial blood glucose levels were measured after 50 g oral glucose load and after the consumption of a portion of different foods containing 50 g of carbohydrates. We also evaluated the associations between postprandial glucose responses and the clinical characteristics of the participants at the beginning of the study. Methods: 12 healthy volunteers (age: 36 ± 17 years, BMI: 24.9 ± 3.5 kg/m^2^) ate white bread (WB) and whole grain (WG) bread and drank a 50 g glucose drink as reference. Postprandial glucose responses were evaluated by CGM, IV and C blood glucose measurements. Incremental area under the curve (AUC_i_) of postprandial blood glucose was calculated for 1 h (AUC_i 0-60_) and 2 h (AUC_i 0-120_). Results: After the consumption of white bread and whole grain bread, the AUC_i 0-60 min_ did not differ between CGM and IV or C. AUC_i 0-120 min_ of CGM showed no difference compared to C. Correlation analyses revealed a positive association of age with glucose AUC_i 0-120_ (*r* = 0.768; *P* = 0.004) and WG AUC_i 0-120_ (*r* = 0.758; *P* = 0.004); fasting blood glucose correlated with WG AUC_i 0-120_ (*r* = 0.838; *P* < 0.001). Conclusion: Despite considerable inter-individual variability of postprandial glycemic responses, CGM evaluated postprandial glycemic excursions which had comparable results compared to standard blood glucose measurements under real-life conditions. Associations of AUC_i 0-60_ and AUC_i 0-120_ postprandial glucose response with age or fasting blood glucose could be shown.

## 1. Introduction

Self-monitoring of blood glucose (SMBG) levels in capillary blood samples is still the most widely-used method for the evaluation of glucose control in patients with glucometabolic diseases [1]. Blood glucose measurement evaluates foods according to their impact on postprandial glycemic excursions [2]. It is of interest to note that the postprandial response to a definite foodstuff shows a high inter- and intra-individual variability [3]. Parts of this variability are also be caused by methodological issues [4]. However, despite its importance, particularly when considering increasing prevalence rates of impaired glucose tolerance and diabetes [5], no method exists for determining and predicting an individual postprandial blood glucose response to food [6]. Compared to standard measurement methods, such as capillary or venous blood glucose testing, continuous glucose monitoring (CGM) might be able to conduct a more comprehensive glycemic assessment and overcomes some of the shortcomings of the other methods [7]. This advantage could allow an individual and comprehensive evaluation of daily glycemic excursions.

The aim of the present study was (i) to examine whether CGM allows for the appropriate evaluation of postprandial blood glucose levels under real-life conditions and to (ii) investigate the association between different parameters of glycemic responses with descriptive parameters of the study subjects.

## 2. Materials and Methods

### 2.1. Study Population

Twelve healthy eligible male volunteers (inclusion criteria: ≥18 years old; exclusion criteria: acute diseases, severe illness with in-patient treatment during the last 3 months, weight change >2 kg/week during the last month, smoking cessation during the last 3 months, drugs for active weight reduction, chronic medication, fasting blood glucose >125 mg/dL (diabetes)) were included. The first participant was enrolled on 1 Dec 2018 and the last participant finished the study on 31 May 2019. The study was conducted at the West-German Centre of Diabetes and Health (WDGZ) in Düsseldorf, Germany, in accordance with the ethical standards laid down in the 1964 Declaration of Helsinki and its later amendments. The research protocol was approved by the ethics committee of the “Ärztekammer Nordrhein”, Düsseldorf (No. 2017409). All participants gave written informed consent prior to their inclusion into the study.

### 2.2. Foodstuff

Two products were studied: white bread (Butter Toast^®^, Golden Toast, Wittenberg, Germany) and whole grain bread (1688 Mehrkorn^®^, Harry-Brot, Schenefeld, Germany). One portion (containing 50 g digestible carbohydrates) was eaten immediately before the beginning of the test in the morning after an overnight fast of at least 10 h. Before testing, participants ate as usual on the previous day without a standard meal and refrained from consuming alcohol and exercising for 72 h. A 200-mL glucose drink (Accu-Chek Dextrose O.G.-T. Saft^®^, Roche Diabetes Care, Mannheim, Germany), containing also 50 g of carbohydrates, was used as the reference product. Energy and macronutrient distribution of foods are shown in Table S1.

### 2.3. Study Design

One day prior to the study beginning, participants were equipped with a CGM system (FreeStyle Libre^®^, Abbott Diabetes Care, Alameda, CA, USA), i.e., the glucose sensor of this system was attached to the upper arm. This CGM system provides glucose recordings every 15 min over a period of 14 days. The glucose data were downloaded manually by a scan with a handheld device. The sensor accuracy of 73.2% had been determined using ISO 15197:2013 (= percentage of sensor values that are within 0.8 mmol/L of the reference value at glucose concentrations <5.6 mmol/L and within 15% at glucose concentrations ≥5.6 mmol/L) [8]. The mean absolute relative difference (MARD) between sensor and venous glucose measurements was 13.2 ± 10.9% [8]. Other methods of determination for the accuracy of CGM confirm strong correlations (*r^2^* = 0.90) with venous blood glucose levels [9] as well as capillary blood glucose levels [10].

On the second day of the study, participants consumed portions of these three test products containing 50 g of available carbohydrate on 3 separate days without a washout period. The products were tested in random order at the same time of the morning after a 10 h overnight fast. Venous blood samples (IV) were collected at 0, 15, 30, 45, 60, 75, 90, 105 and 120 min postprandial by inserting an intravenous cannula into a forearm vein. Capillary blood samples (C) were obtained through finger pricking. Blood glucose levels in these samples were measured with a high quality SMBG system (ContourXT/Contour next, Ascensia Diabetes Care, Leverkusen, Deutschland, Germany).

### 2.4. Measurements

Participants visited the WDGZ at the first day of the study for determination of anthropometric measurements and clinical data comprising age, body weight, height, body mass index (BMI), waist circumference and fat mass. Blood samples for measurement of total cholesterol, high-density lipoprotein (HDL) cholesterol, low-density lipoprotein (LDL) cholesterol, triglycerides, hemoglobin A1c (HbA1c), fasting blood glucose, and fasting plasma insulin were taken as well. Body weight was measured in light clothing to the closest 0.1 kg, height to the closest 0.5 cm, and waist circumferences at the minimum abdominal girth (midway between the rib cage and the iliac crest). Fat mass (FM) was determined by bioelectrical impedance analysis with multi frequency measuring using a body composition scale (Seca mBCA515, Seca, Hamburg, Germany). Venous blood samples were collected for the determination of laboratory parameters and were analyzed at the local laboratory with intra-assay coefficients of variability (CV) of 1.6% for HbA1c, 1.9% for fasting blood glucose, 3.6% for insulin, 2.8% for HDL cholesterol, 3.3% for LDL cholesterol, 2.6% for total cholesterol, and 2.9% for triglycerides [11]. To protect venous blood samples from hemolysis, S-Monovette^®^ tubes (SARSTEDT, Nümbrecht, Germany) were used [12].

### 2.5. Calculation of Glycemic Variables

The incremental AUC (AUC_i_) of postprandial glucose excursions was calculated geometrically as the sum of the areas of the triangles and trapezoids over 2 h, excluding the area below the initial fasting glucose concentration [13].

### 2.6. Statistical Analysis

Data are presented as arithmetic means and standard deviations (mean ± SD). Moreover, data which were not normally distributed were analyzed with Mann-Whitney U or Wilcoxon and Spearman rank correlation test to determine differences between measurement procedures and to determine correlations between variables. Postprandial blood glucose excursions of a glucose drink, white bread and whole grain bread were analyzed after 60 and 120 min.

Multiple linear regression analyses were performed in which AUC_i_ had been set as a dependent variable and age, BMI, fasting blood glucose, HbA1c, and fasting plasma insulin levels as independent variables. All statistical tests were two sided, and the level of significance was set at α = 0.05. All analyses were performed using SPSS 22.0 (SPSS Inc., Chicago, IL, USA) and GraphPad Prism 6.04 (GraphPad Software, San Diego, CA, USA).

## 3. Results

**Figure 1:**
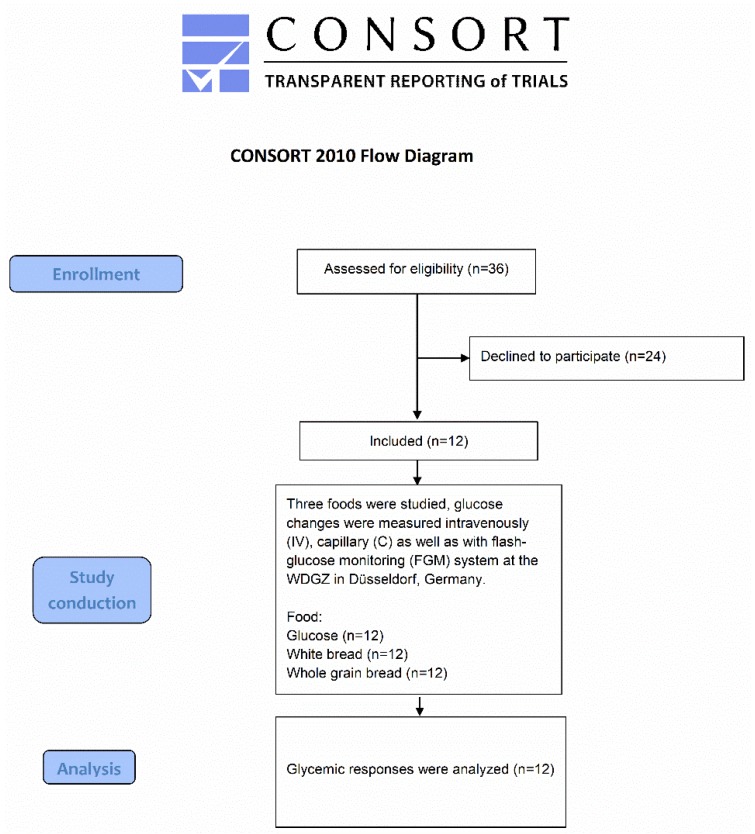
Consort flow diagram.

Study population. All participants finished the study (Table 1, Figure 1) and no adverse effects were reported.

**Figure 2:**
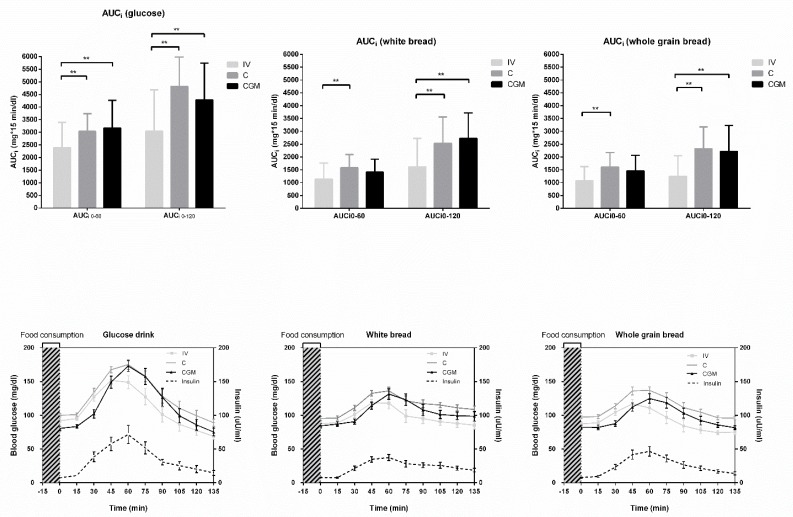
Postprandial glucose responses as AUC_i 0-60_ and AUC_i 0-120_ after (**a**) glucose, (**b**) white bread or (**c**) whole grain bread consumption and as absolute values after (**d**) glucose, (**e**) white bread, and (**f**) whole grain bread consumption. IV, intravenously; C, capillary; CGM, continuous glucose monitoring. Shown are means ± SD; ** *P* < 0.01.

Fasting glucose levels and postprandial glycemic excursions. Fasting blood glucose values at t = 0 min differed significantly between the three glucose measurements (IV: 91 ± 10 mg/dL; C: 98 ± 9 mg/dL; CGM: 84 ± 12 mg/dL; all *P* < 0.001). Postprandial glucose excursions in the first 60 min after drinking the glucose drink (AUC_i 0-60min_) as well as in the first 120 min (AUC_i 0-120min_) were significantly higher in C and CGM compared to IV (all *P* < 0.01). After the consumption of white bread and whole grain bread the AUC_i 0-60min_ did not differ between CGM and IV but between C and IV (*P* < 0.01). However, AUC_i 0-120min_ was significantly higher in C and CGM vs. IV (both *P* < 0.01, Figure 2a–c). Absolute postprandial glucose changes are shown in Figure 2d–f.

Correlation analyses: Age was positively correlated with the AUC_i 0-60_ (*r* = 0.629; *P* = 0.029) and AUC_i 0-120_ (*r* = 0.768; *P* = 0.004) after the glucose drink (Table S2). This correlation also existed for whole grain bread (AUC_i 0-60_: *r* = 0.867; *P* < 0.001; AUC_i 0-120_: *r* = 0.758; *P* = 0.004). Fasting blood glucose correlated positively with AUC_i 0-120_ after the glucose drink (*r* = 0.620; *P* = 0.032), whole grain bread AUC_i 0-60_ (*r* = 0.838; *P* < 0.001) and whole grain bread AUC_i 0-120_ (*r* = 0.838; *P* < 0.001).

## 4. Discussion

The usage of a CGM system for the evaluation of postprandial glycemic excursions in a real-world-setting resulted in comparable results as opposed to using the standard blood glucose measurement capillary glucose testing. Since CGM provides more data than serial blood glucose measurements, it allows for a more comprehensive glycemic assessment (i.e., the most notable benefit of CGM is the wealth of time-series glucose data revealing real-time temporal trends and patterns and the ability to announce if glucose levels are detected above or below a specific threshold) [14]. Furthermore, CGM overcomes some drawbacks of blood glucose measurements (e.g., user error on test accuracy, the need for multiple fingerstick blood samples each day, and the limited data available (only a single snap shot of glucose concentrations and not trending data) [14,15]), and has been evaluated in patients without [16] and with type 1 or type 2 diabetes [7,17].

As food items with fast absorbable carbohydrates induce a rapid increase in postprandial glucose excursions, AUC_i_ was calculated for the 1^st^ hour and 2 h after starting eating. There were significant correlations of AUC_i_ in the first 60 min with the glucose drink and whole grain bread as well as a strong correlation of AUC_i_ in the 120 min with whole grain bread with the independent variables age and fasting blood glucose. Earlier studies have shown that particularly age [18] as well as sex are strong influencing factors. Furthermore, fasting blood glucose correlated with the 1 h and 2 h values of postprandial blood glucose response. Further factors that may affect postprandial glucose responses comprise genetics [19], lifestyle [20], exocrine pancreatic and glucose transporters activity levels [21], as well as gut microbiota [6].

Certain strengths and limitations of our study have to be considered. Only men were included in the study to avoid gender-specific influences, especially with regard to the strong impact of the menstrual cycle and oral contraceptives on postprandial glucose excursions [22] or differences in glucose clearance between males and females and potential deviations in fat oxidation. Therefore, the findings might not be generalizable or transferable to other cohorts, particularly with metabolic impairments like diabetes. However, the real-life study approach indicates that CGM could be a pragmatic alternative for individual everyday usage to determine postprandial glucose changes. A further limitation could be the low sample size (*n* = 12). The lack of power might have led to no differences among methods. In future studies, more complex analyses methods could be used to analyze longitudinal data in more detail, applying mixed effect models considering complex correlation structures, irregularly spaced visits, missing data, and mixtures of time-varying and static covariate effects. There are also differences in fasting blood glucose levels between the measurements. This difference is probably caused by the different fluids (venous, capillary and subcutaneous) taken from different compartments during blood sampling [9].

The major strength of this study is the innovative approach to determine individual glycemic responses for common carbohydrate-containing foods based on a CGM system. As postprandial glycemic response has strong inter-subject variability, our approach makes it possible that every person can determine their individual glycemic responses by using CGM systems, which could be an important step for the management of blood glucose levels. Although CGM systems were primarily developed for patients with diabetes, the present findings indicate potential benefits and application possibilities, even for healthy people, for educative purposes to adapt eating and moving habits, aiming to keep blood glucose levels within a normal range. Moreover, real-time information regarding glucose can be useful in the case of nutrition-related uncertainties. Furthermore, CGM curves could also be used in future studies to determine individual glycemic indices, especially when considering the inter-individual variability of postprandial glucose responses [6].

The present study points towards a useful application of CGM systems for the determination of individual postprandial glycemic responses under everyday life conditions.

## Figures and Tables

**Table 1:** Baseline characteristics.

	Study Group (*n* = 12)
Age (years)	36 ± 17
Weight (kg)	84 ± 12
BMI (kg/m^2^)	24.9 ± 3.5
Waist circumference (cm)	89 ± 12
Fat mass (%)	26 ± 6
Fat mass (kg)	20 ± 8
HbA_1c_ (%) (mmol/mol)	5.3 ± 0.5 (34.1 ± 5.4)
Fasting blood glucose (mg/dL) (mmol/L)	93 ± 8 (5.1 ± 0.5)
Fasting plasma insulin (uU/mL) (pmol/L)	7.5 ± 4.0 (54.1 ± 28.4)
HOMA-IR	1.7 ± 0.1
Total cholesterol (mg/dL) (mmol/L)	165 ± 36 (4.3 ± 0.9)
HDL (mg/dL) (mmol/L)	53 ± 13 (1.4 ± 0.3)
LDL (mg/dL) (mmol/L)	105 ± 38 (2.7 ± 1.0)
Triglyceride (mg/dL) (mmol/L)	119 ± 62 (1.3 ± 0.7)

Shown are means ± standard deviations; BMI, body mass index; HbA_1c_, glycated hemoglobin A; HDL, high-density-lipoprotein; HOMA-IR, homeostatic model assessment—insulin resistance; LDL, low-density-lipoprotein.

## Supplementary Materials

The following are available online at https://www.mdpi.com/2072-6643/11/10/2305/s1, Table S1: Energy and macronutrient distribution of foods, Table S2: Correlation matrix between AUC_i_ and selected patient and baseline characteristics.
