# Supplementary material for: Determination of Postprandial Glycemic Responses by Continuous Glucose Monitoring in a Real-World Setting
Source: Nutrients. 2019 Sep 27;11(10):2305. doi: 10.3390/nu11102305 (PMC6835966; doi:10.3390/nu11102305)
Supplement: Supplementary file 1 [file nutrients-11-02305-s001.pdf]

Table S1. Energy and macronutrient distribution of foods.

	**Glucose drink**		**White bread**		**Whole grain bread**	
	**100 g**	**Portion (67 g)**	**100 g**	**portion (104 g)**	**100 g**	**portion (143 g)**
Energy (kcal)	1256	837	1120	1167	962	1374
Energy (kcal)	300	200	265	276	229	327
Available carbohydrate (g)	75	50	48	50	35	50
Sugar (g)	75	50	4.0	4.2	2.3	3.3
Total fiber (g)	0	0	3.0	3.1	6.9	9.9
Total fat (g)	0	0	3.8	4.0	4.9	7.0
Saturated fatty acids (g)	0	0	2.0	2.1	0.8	1.1
Total protein (g)	0	0	8.2	8.5	7.7	11
Sodium (mg)	0	0	1.1	1.1	1.2	1.7

Data are based on nutritional information from every product used per 100 g product as well as per portion consumed.

Table S2. Correlation matrix between AUC_i and selected patient and baseline characteristics.

**Parameter**	**$AUC_{i\ G\ 0-60}$**	**$AUC_{i\ G\ 0-120}$**	**$AUC_{i\ WG\ 0-120}$**	**$AUC_{i\ WG\ 0-120}$**
Age (yrs)	r = 0.629<br>P =0.029	r = 0.768<br>P =0.004	**r = 0.867**<br>**P <0.001**	r = 0.758<br>P =0.004
BMI (kg/m²)	r = 0.196<br>P =0.542	r = 0.364<br>P =0.245	r = 0.350<br>P =0.265	r = 0.182<br>P =0.572
Fasting blood glucose (mg/dl)	r = 0.444<br>P =0.149	r = 0.620<br>P =0.032	**r = 0.838**<br>**P <0.001**	**r = 0.838**<br>**P <0.001**
HbA1c (%)	r = 0.263<br>P =0.409	r = 0.418<br>P =0.177	r = 0.319<br>P =0.312	r = 0.270<br>P =0.396
Fasting plasma insulin (µU/mL)	r =- 0.084<br>P =0.795	r = 0.042<br>P =0.897	r = 0.105<br>P = 0.746	r = 0.056<br>P =0.863

Bold numbers are significant after Bonferroni correction ($P < 0.003125$). G, glucose; WG, whole grain bread.
